# Supplementary material for: PEX3 promotes regenerative repair after myocardial injury in mice through facilitating plasma membrane localization of ITGB3
Source: Commun Biol. 2024 Jul 1;7:795. doi: 10.1038/s42003-024-06483-0 (PMC11217276; doi:10.1038/s42003-024-06483-0)
Supplement: Supplementary file 2 — Supplemental Materials [file 42003_2024_6483_MOESM2_ESM.pdf]

PEX3 promotes regenerative repair after myocardial injury in mice through facilitating plasma membrane localization of ITGB3

Jia-Teng Sun[#], Zi-Mu Wang[#], Liu-Hua Zhou[#], Tong-Tong Yang[#], Di Zhao, Yu-Lin Bao, Si-Bo Wang, Ling-Feng Gu, Jia-Wen Chen, Tian-Kai Shan, Tian-Wen Wei, Hao Wang, Qi-Ming Wang, Xiang-Qing Kong, Yong Ji, Li-Ping Xie, Ai-Hua Gu, Yang Zhao, Feng Chen, Yi-Qiang Cui[*], and Lian-Sheng Wang[*]

# These authors contributed equally.
* Corresponding authors.

Supplemental Figure and Figure legends

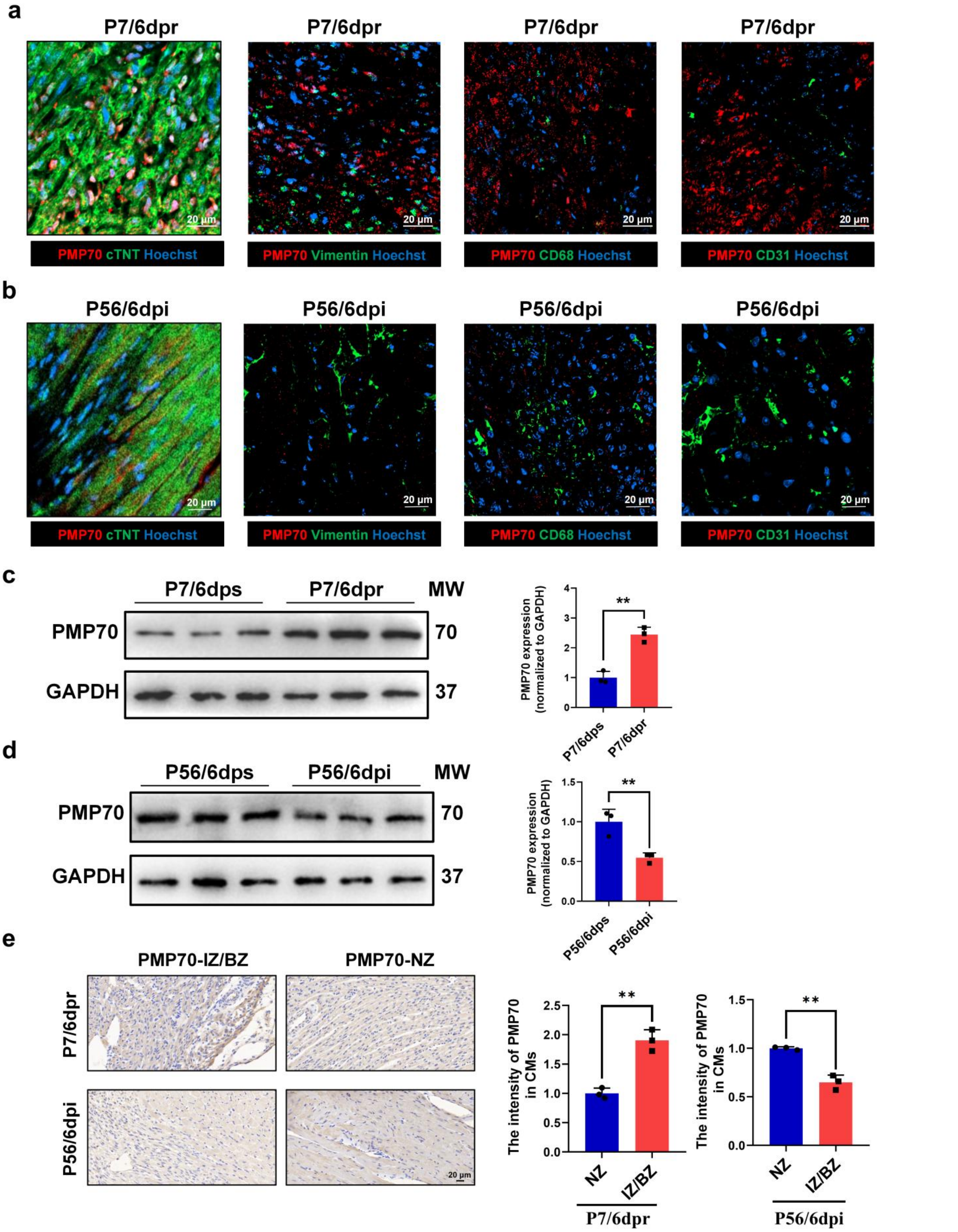

Supplemental Figure 1: a. Representative images of PMP70 (red) at P7/6dpr of mice heart. cTNT (Cardiomyocyte) /Vimentin (Fibroblast) /CD68 (Macrophage) /CD31 (Endothelial cell) =Green, Hoechst=Blue. Scale bar=20 μm. **b.** Representative images of PMP70 (red) at P56/6dpi of mice heart. cTNT (Cardiomyocyte) /Vimentin (Fibroblast) /CD68 (Macrophage) /CD31 (Endothelial cell) =Green, Hoechst=Blue. Scale bar=20 μm. **c.** Western blotting and quantification analysis of PMP70 in P7/6dps and P7/6dpr hearts (n=3 mice per group). **d.** Western blotting and

quantification analysis of PMP70 at P56/6dps and P56/6dpi hearts (n=3 mice per group). **e.** Representative and quantification IHC images of PMP70 in the injury zone/border zone and normal zone in P7/6dpr and P56/6dpi mice (n=3 mice per group). Scale bar=20 μm. Unpaired t test applied for c-e. Data shown as mean±SEM. **, P<0.01.

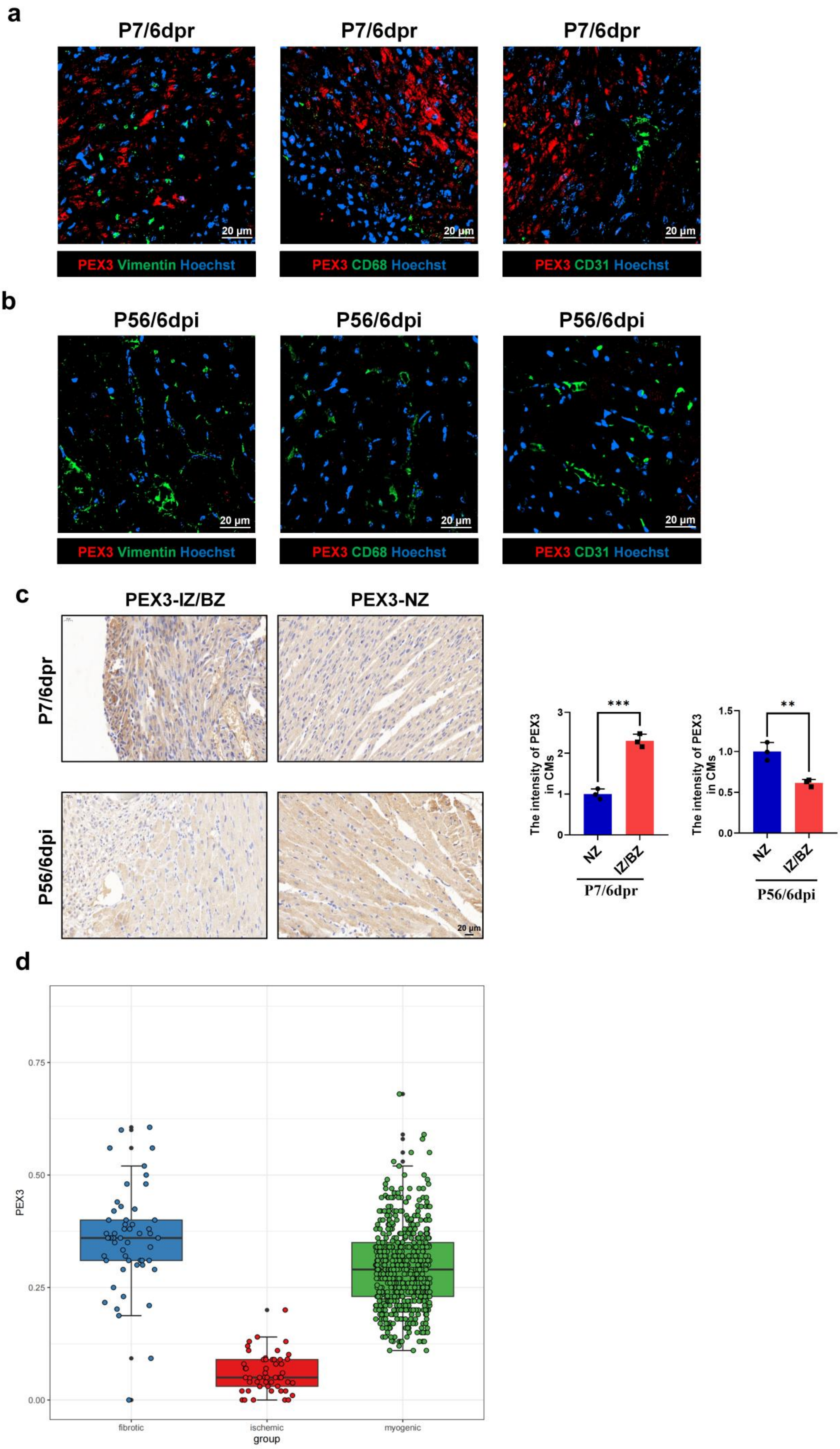
a
P7/6dpr
P7/6dpr
P7/6dpr
20 µm
PEX3 Vimentin Hoechst
PEX3 CD68 Hoechst
PEX3 CD31 Hoechst
b
P56/6dpi
P56/6dpi
P56/6dpi
20 µm
PEX3 Vimentin Hoechst
PEX3 CD68 Hoechst
PEX3 CD31 Hoechst
c
PEX3-IZ/BZ
PEX3-NZ
P7/6dpr
P56/6dpi
20 µm
The intensity of PEX3 in CMs
***
**
NZ
IZ/BZ
P7/6dpr
P56/6dpi
d
PEX3
0.00
0.25
0.50
0.75
fibrotic
ischemic
myogenic
group

Supplemental Figure 2: a. Representative images of PEX3 (red) at P7/6dpr of mice heart. Vimentin (Fibroblast) /CD68 (Macrophage) /CD31 (Endothelial cell) =Green, Hoechst=Blue. Scale bar=20 µm. **b.** Representative images of PEX3 (red) at P56/6dpi of mice heart. Vimentin (Fibroblast) /CD68 (Macrophage) /CD31 (Endothelial cell) =Green, Hoechst=Blue. Scale bar=20 µm. **c.** Representative and quantification IHC images of PEX3 in the injury zone/border zone and normal zone in P7/6dpr and P56/6dpi mice (n=3 mice per group). Scale bar=20 µm. **d.** Using Metacell's method to detect expression of PEX3 in myogenic, ischemic and fibrotic areas by the publicly available single-cell nuclear sequencing data in human myocardial infarction. Unpaired t test applied for c. Data shown as mean±SEM. **, P<0.01, ***, P<0.001.

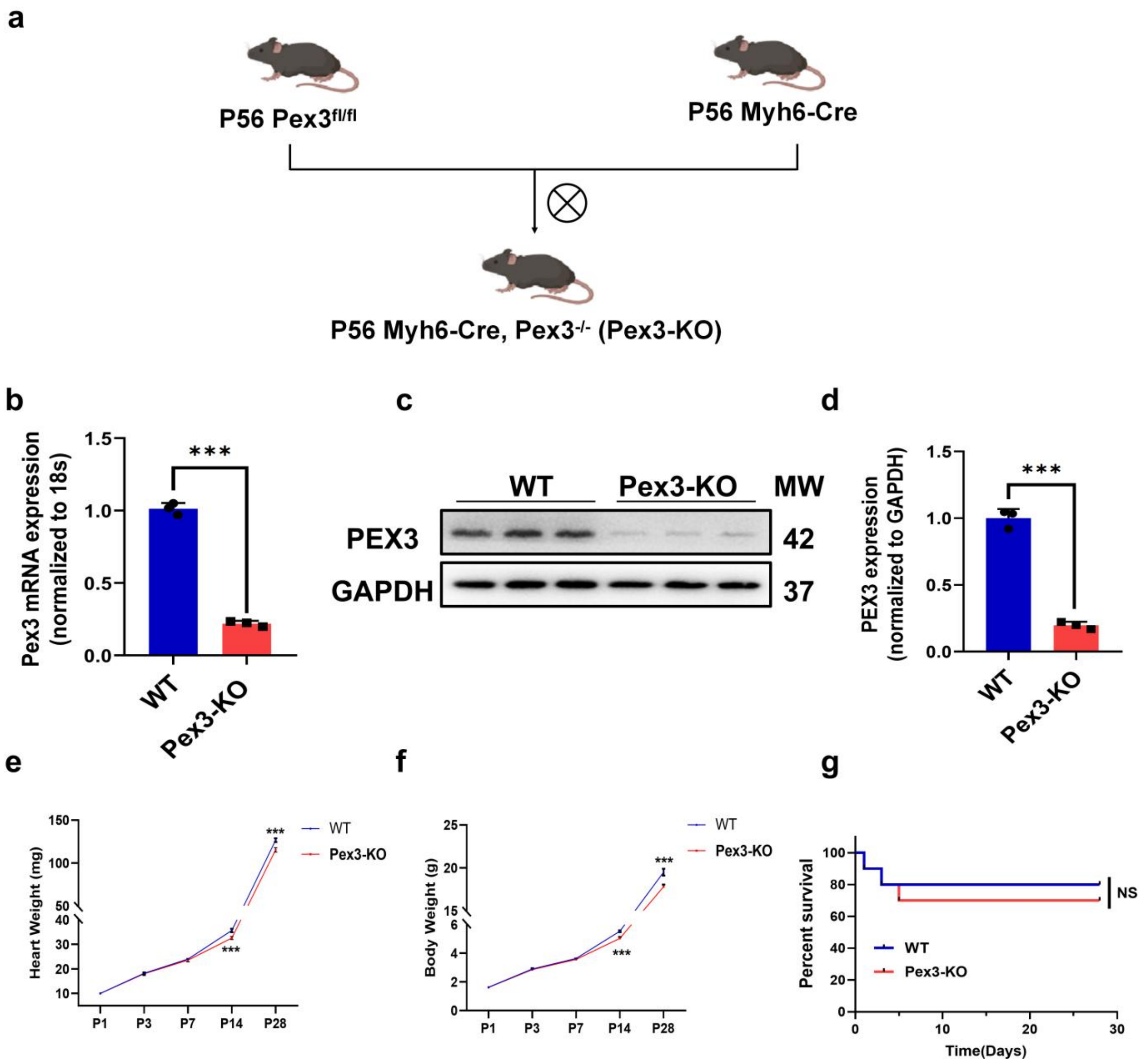

Supplemental Figure 3: a. The schematic illustration for constructing and reproduction Pex3-KO mice. **b.** mRNA expression level of PEX3 in the WT and Pex3-KO mice hearts. The fold change was calculated by normalizing to 18S (n=3 mice per group). **c-d.** Western blotting and quantification analysis of PEX3 in WT and Pex3-KO mice hearts (n=3 mice per group). **e-f.** Heart weight and body weight of WT and Pex3-KO mice from P1 to P28 (n=6 mice per group). **g.** Survival rate of WT and Pex3-KO mice from P1 to P28 (n=10 mice per group). Unpaired t test applied for b, d. Two-way ANOVA and Tukey's Multiple Comparison Test were performed for e-f. Kaplan-Meier (log-rank test) was performed for g. Data shown as mean±SEM. N.S, Not Significant, ***, P<0.001.

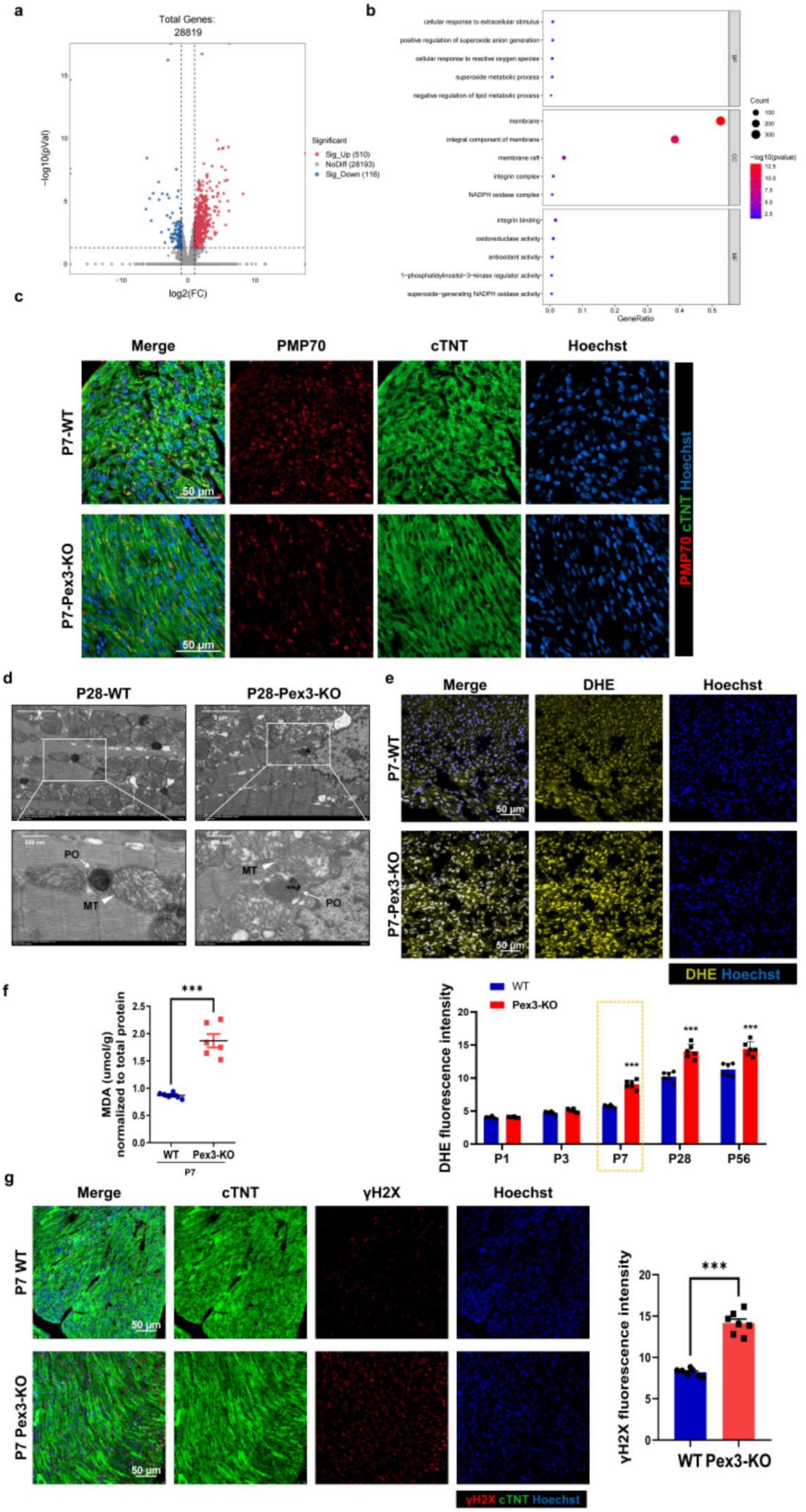
a
Total Genes:
28819
-log10(pVal)
log2(FC)
Significant
Sig_Up (510)
NoDiff (28193)
Sig_Down (116)
b
cellular response to extracellular stimulus
positive regulation of superoxide anion generation
cellular response to reactive oxygen species
superoxide metabolic process
negative regulation of lipid metabolic process
membrane
integral component of membrane
membrane raft
integrin complex
NADPH oxidase complex
integrin binding
oxidoreductase activity
antioxidant activity
1-phosphatidylinositol-3-kinase regulator activity
superoxide-generating NADPH oxidase activity
GeneRatio
Count
-log10(pvalue)
c
Merge
PMP70
cTNT
Hoechst
P7-WT
P7-Pex3-KO
50 μm
PMP70 cTNT Hoechst
d
P28-WT
P28-Pex3-KO
PO
MT
e
Merge
DHE
Hoechst
P7-WT
P7-Pex3-KO
50 μm
DHE Hoechst
f
MDA (umol/g) normalized to total protein
WT
Pex3-KO
P7
***
WT
Pex3-KO
DHE fluorescence intensity
P1
P3
P7
P28
P56
g
Merge
cTNT
γH2X
Hoechst
P7 WT
P7 Pex3-KO
50 μm
γH2X cTNT Hoechst
γH2X fluorescence intensity
WT Pex3-KO

Supplemental Figure 4: a. Volcano plot resulting from the RNA-seq analysis showing upregulated (Red) and downregulated (Blue) genes in WT and Pex3-KO mice hearts. Genes were considered significant if P value < 0.05 after false discovery rate test, and differentially expressed if $|\log2(FC)| \geq 1$ (n=3 per group). **b.** Gene Ontology analysis of differential expression genes in WT and Pex3-KO mice hearts. $|\log2(FC)| \geq 1$, $P < 0.05$. **c.** Representative images of WT and Pex3-KO mice myocardium stained with PMP70 (red), cTNT (green) and Hoechst (blue) at P7. Scale bar=50 µm. **d.** Representative transmission electron microscopy images of peroxisome function and structure in WT and Pex3-KO mice at P28. PO indicates peroxisome, MT indicates mitochondria. Scale bar=500 nm, scale bar=2 µm. **e.** IF staining and quantification analysis of DHE (yellow) in WT and Pex3-KO mice from P1 to P56 (n=6 mice per group). Hoechst=Blue. Scale bar=50 µm. **f.** The level of Malondialdehyde between WT and Pex3-KO mice at P7 (n=6 mice per group). **g.** IF staining and quantification analysis of γH2X (red) between WT and Pex3-KO mice at P7 (n=7 mice per group). cTNT=Green, Hoechst=Blue. Scale bar=50 µm. Unpaired t test applied for f-g. Two-way ANOVA and Tukey's Multiple Comparison Test were performed for e. Data shown as mean±SEM. ***, P<0.001.

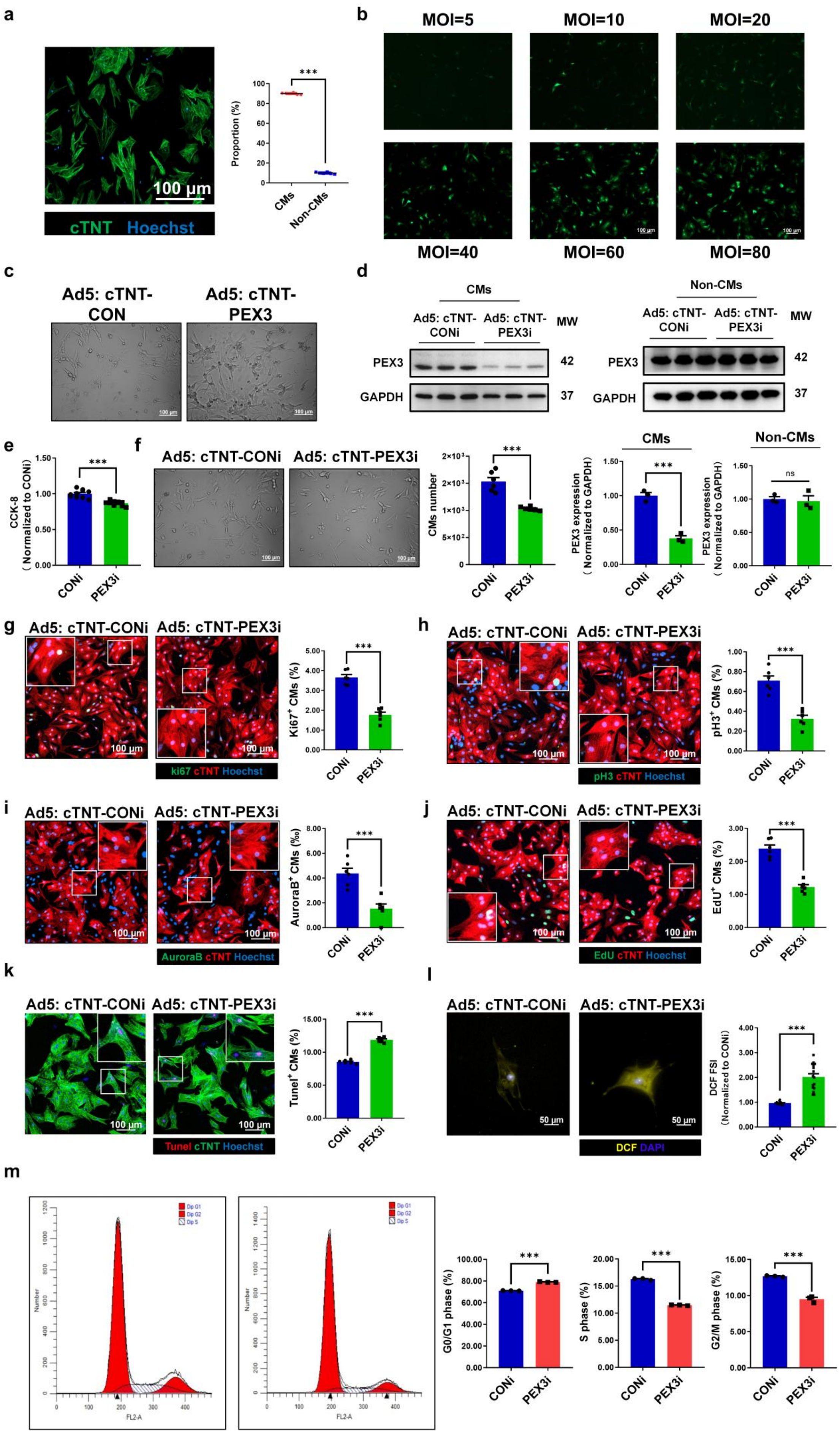

a
cTNT Hoechst
100 μm
Proportion (%)
CMs
Non-CMs
b
MOI=5
MOI=10
MOI=20
MOI=40
MOI=60
MOI=80
c
Ad5: cTNT-CON
Ad5: cTNT-PEX3
d
CMs
Non-CMs
Ad5: cTNT-CONi
Ad5: cTNT-PEX3i
MW
PEX3
GAPDH
42
37
e
CCK-8 (Normalized to CONi)
f
CMs number
PEX3 expression (Normalized to GAPDH)
ns
CONi
PEX3i
g
ki67 cTNT Hoechst
Ki67+ CMs (%)
h
pH3 cTNT Hoechst
pH3+ CMs (%)
i
AuroraB cTNT Hoechst
AuroraB+ CMs (‰)
j
EdU cTNT Hoechst
EdU+ CMs (%)
k
Tunel cTNT Hoechst
Tunel+ CMs (%)
l
DCF DAPI
50 μm
DCF FSI (Normalized to CONi)
m
G0/G1 phase (%)
S phase (%)
G2/M phase (%)

Supplemental Figure 5: a. Purity of isolated P1 neonatal primary cardiomyocytes or non-CMs revealed by immunofluorescence analysis of the CM marker cTnT (n=6 per group). Scale bar=100 μm. **b.** Representative images of primary cardiomyocytes and transfected with adenoviruses carrying GFP-tagged PEX3 in different MOIs. Scale bar=100 μm. **c.** Representative images of CMs number in Ad5-cTNT-PEX3 groups and Ad5-cTNT-CON groups. Scale bar=100 μm. **d.** Western blotting and quantitative analysis of PEX3 protein levels in primary CMs and Non-CMs tranfected with Ad5-cTNT-PEX3i and Ad5-cTNT-CONi (n=3 per group). **e.** CMs viability quantified between Ad5-cTNT-PEX3i groups and Ad5-cTNT-CONi groups by CCK-8 assay (n=10 per group). **f.** The total number of CMs and representative images between Ad5-cTNT-PEX3i groups and Ad5-cTNT-CONi groups (n=6 per group). Scale bar=100 μm. **g-j.** IF staining and quantification analysis of Ki67, pH3, Aurora B and EdU (green) after transfection with Ad5-cTNT-PEX3i or Ad5-cTNT-CONi in primary CMs (n=6 per group). CMs were stained with cTNT (red), nuclei were stained with Hoechst (blue). Scale bar=100 μm. (Ki67: 3837 CMs in the Ad5-cTNT-CONi group, 3758 CMs in the Ad5-cTNT-PEX3i group; pH3: 3283 CMs in the Ad5-cTNT-CONi group, 3601 CMs in the Ad5-cTNT-PEX3i group; Aurora B: 3373 CMs in the Ad5-cTNT-CONi group, 3145 CMs in the Ad5-cTNT-PEX3i group; EdU: 4371 CMs in the Ad5-cTNT-CONi group, 3970 CMs in the Ad5-cTNT-PEX3i group). **k.** IF staining and quantification of Tunel (red) in the PEX3i groups and CONi groups post O/GD treatment (n=6 per group). CMs were stained with cTNT (green), nuclei were stained with Hoechst (blue). Scale bar=100 μm. (Tunel: 3372 CMs in the Ad5-cTNT-CONi group, 3572 CMs in the Ad5-cTNT-PEX3i group). **l.** IF staining and quantification analysis of DHE (yellow) in PEX3i groups compared with CONi groups (n=6 per group). Nucleus was stained with DAPI (blue). Scale bar=50 μm. **m.** Flow cytometry analysis of primary CMs transfected with Ad5-cTNT-PEX3i or Ad5-cTNT-CONi (n=3 per group). Unpaired t test applied for a, d-m. Data shown as mean±SEM. N.S, Not Significant, ***, P<0.001.

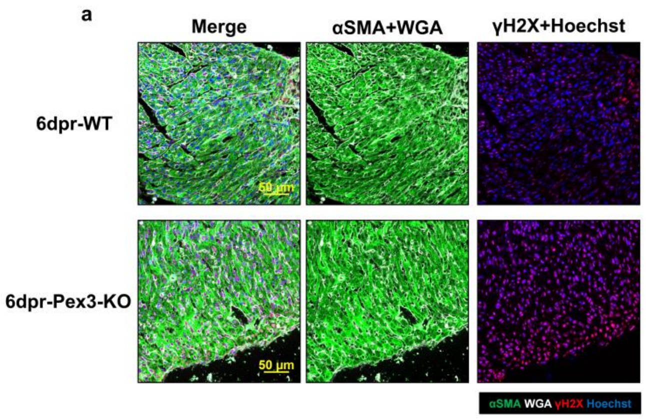

Supplemental Figure 6: a. IF staining analysis of γH2X (red) between WT mice and Pex3-KO mice at 6dpr. Cell outline was stained with WGA (white), myocardium was stained with α-SMA (green), nuclei were stained with Hoechst (blue). Scale bar=50 μm.

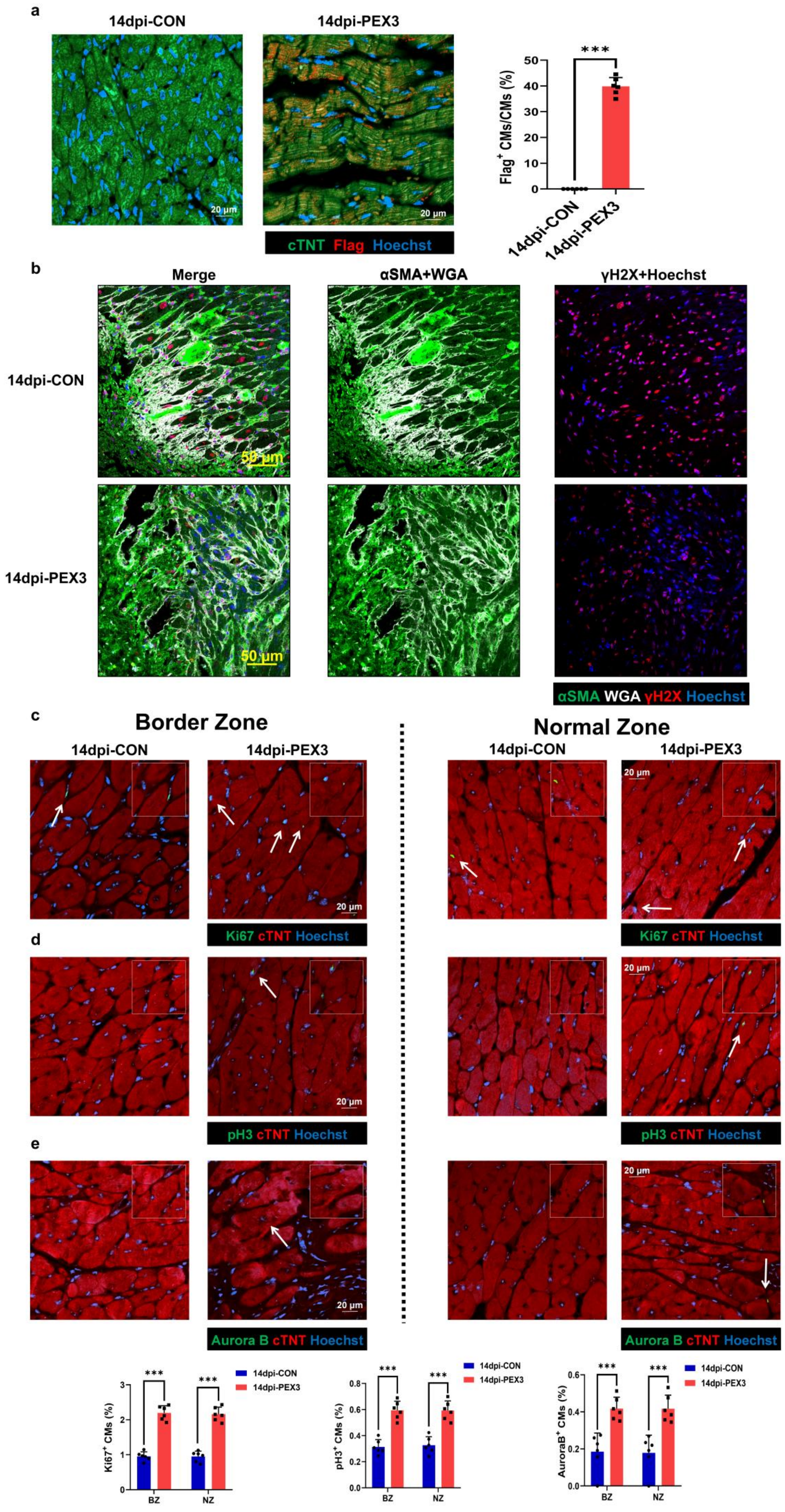

a
14dpi-CON
14dpi-PEX3
20 μm
cTNT Flag Hoechst
***
Flag+ CMs/CMs (%)
b
Merge
αSMA+WGA
γH2X+Hoechst
14dpi-CON
14dpi-PEX3
50 μm
αSMA WGA γH2X Hoechst
c
Border Zone
Normal Zone
Ki67 cTNT Hoechst
d
pH3 cTNT Hoechst
e
Aurora B cTNT Hoechst
Ki67+ CMs (%)
pH3+ CMs (%)
AuroraB+ CMs (%)
BZ
NZ

Supplemental Figure 7: a. IF staining analysis of Flag (red) between CON group and PEX3 group at 14dpi (n=6 mice per group). cTNT=Green, Hoechst=Blue. Scale bar=20 μm. **b.** IF staining analysis of γH2X (red) between CON group and PEX3 group at 14dpi. Cell outline was stained with WGA (white), myocardium was stained with α-SMA (green), nuclei were stained with Hoechst (blue). Scale bar=50 μm. **c-e**. Effects of systemic injection of AAV9-cTNT-PEX3 or AAV9-cTNT-CON 3 days after MI on myocardial cell cycle markers (Ki67, pH3 and Aurora B) (green) in adult mice at 14dpi (n=6 mice per group). cTNT=Red, Hoechst =Blue. Scale bar=20 μm. Unpaired t test applied for a, c-e. Data shown as mean±SEM. ***, P<0.001.

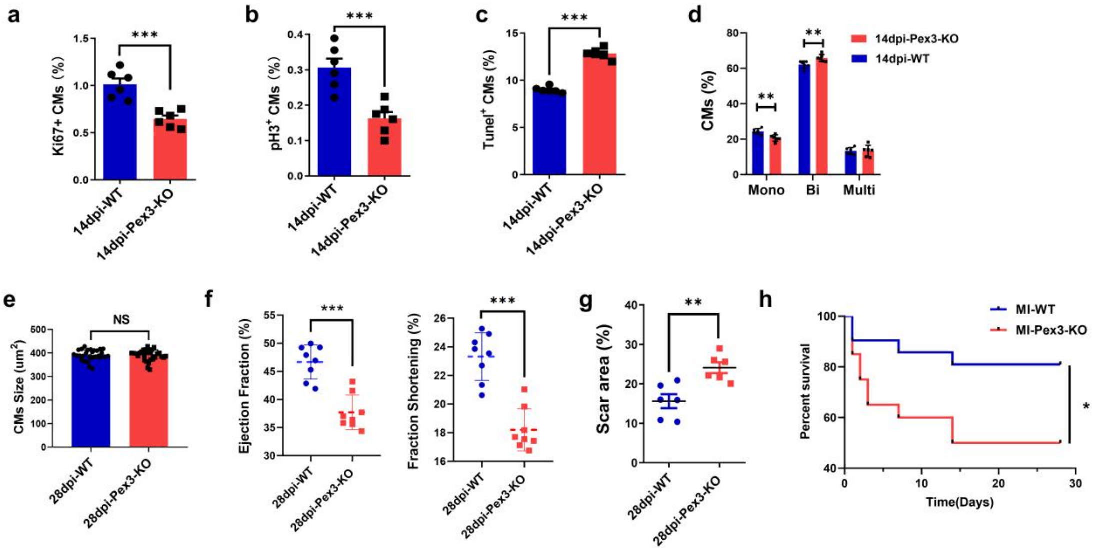

Supplemental Figure 8: a-b. Quantification analysis of Ki67 and pH3 in WT mice compared with Pex3-KO mice at 14dpi (Ki67: 5188 CMs in the WT group, 3868 CMs in the Pex3-KO group; pH3: 4547 CMs in the WT group, 4947 CMs in the Pex3-KO group; n=6 mice per group). **c.** Quantification analysis of Tunel$^+$ signals between WT mice and Pex3-KO mice at 14dpi (Tunel: 4763 CMs in the WT group, 4780 CMs in the Pex3-KO group; n=6 mice per group). **d.** Isolation and quantification of the mononucleated, binucleated and multinucleated cardiomyocytes number between WT mice and Pex3-KO mice at 14dpi (n=6 mice per group). **e.** Quantification analysis of cardiomyocyte size in WT mice and Pex3-KO mice at 28dpi (n=6 mice per group). **f.** Echocardiography measurements of ejection fraction and fractional shortening in WT and Pex3-KO mice at 28dpi (n=8 mice per group). **g.** Quantification of scar tissue in WT mice and Pex3-KO mice at 28dpi (n=6 mice per group). **h.** Survival rate of mice in WT and Pex3-KO mice from 1dpi to 28dpi (n=20-21 mice per group). Unpaired t test applied for a-g. Kaplan-Meier (log-rank test) was performed for h. Data shown as mean±SEM. N.S, Not Significant, *, P<0.05, **, P<0.01, ***, P<0.001.

a

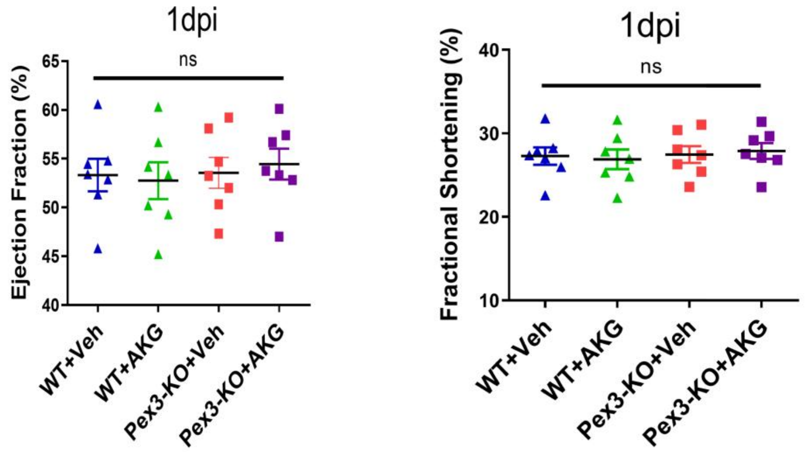

Supplemental Figure 9: a. Echocardiography measurements of ejection fraction and fractional shortening in each group mice after MI at 1dpi (n=7 mice per group). Two-way ANOVA and Tukey's Multiple Comparison Test were performed for a. Data shown as mean±SEM. N.S, Not Significant.

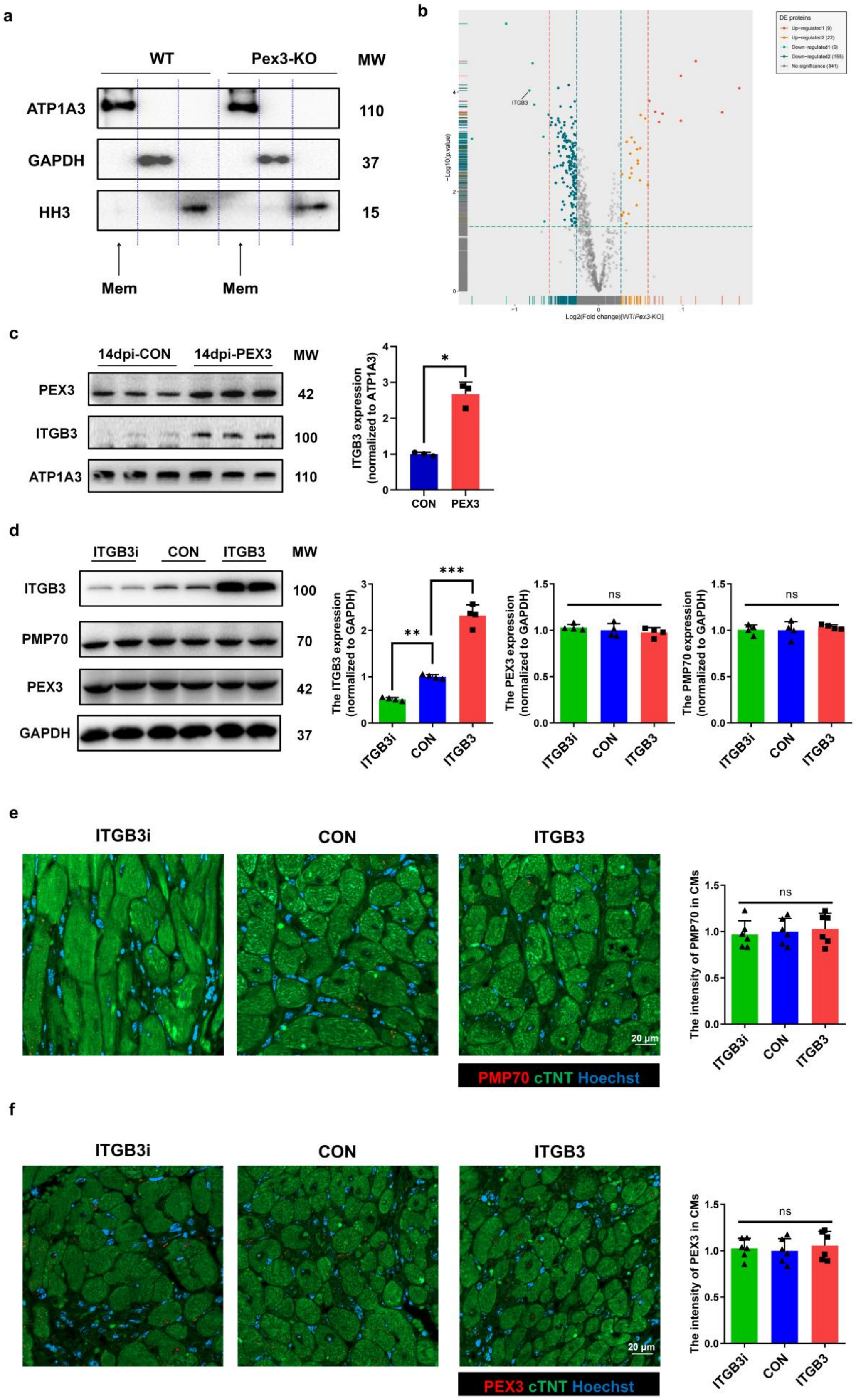

Supplemental Figure 10: a. Isolated the myocardial plasma membrane proteins of P56 WT and Pex3-KO mice and western blotting analysis of ATP1A3, GAPDH and HH3 proteins level. **b.** Volcano plot of the proteomics analysis showing upregulated (Red and Orange) and downregulated (Blue and Green) genes in WT and Pex3-KO adult mice heart. Genes were considered significant if P value < 0.05 after false discovery rate test, and differentially expressed if

FC > 1.2 (n=3 mice per group). **c.** Western blotting and quantification analysis of PEX3 and ITGB3 in adult mice after MI and injected with AAV9-cTNT-PEX3/CON at 14dpi (n=3 mice per group). **d.** Western blotting and quantification analysis of ITGB3, PEX3 and PMP70 in adult mice at 14dpi after MI and AAV9-cTNT-ITGB3 or AAV9-cTNT-ITGB3i injection (n=4 mice per group). **e.** Representative images and quantification analysis of PMP70 (red) in adult mice at 14dpi after MI and AAV9-cTNT-ITGB3 or AAV9-cTNT-ITGB3i injection (n=6 mice per group). cTNT=Green, Hoechst=Blue. Scale bar=20 μm. **f.** Representative images and quantification analysis of PEX3 (red) in adult mice at 14dpi after MI and AAV9-cTNT-ITGB3 or AAV9-cTNT-ITGB3i injection (n=6 mice per group). cTNT=Green, Hoechst=Blue. Scale bar=20 μm. Unpaired t test applied for c-f. Data shown as mean±SEM. N.S, Not Significant, *, P<0.05, **, P<0.01, ***, P<0.001.

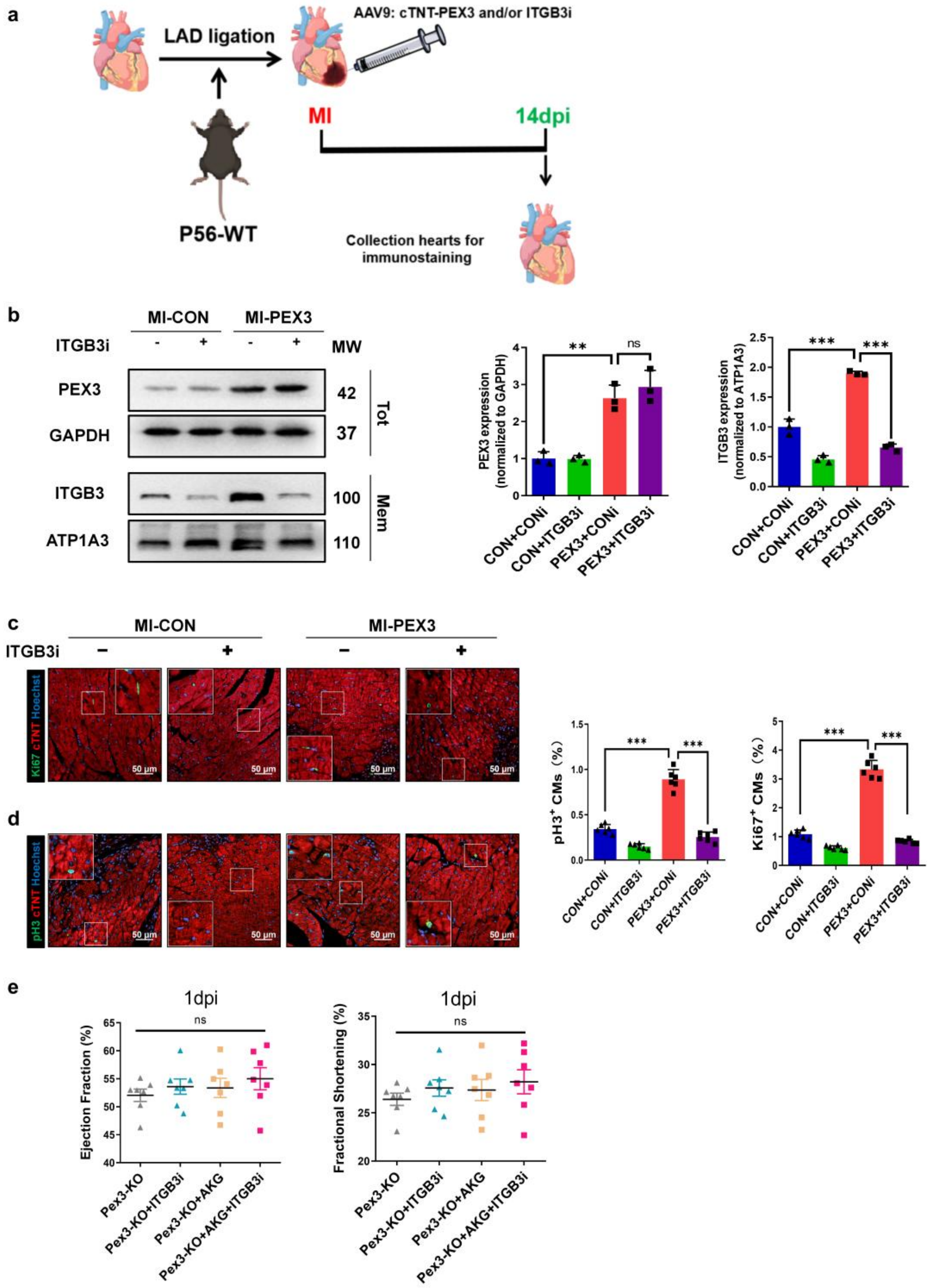

Supplemental Figure 11: a. Schematic illustration of the experimental design. **b.** Western blotting and quantification analysis of PEX3 and ITGB3 in adult mice at 14dpi after MI and AAV9-cTNT-PEX3 (CON) and/or

AAV9-cTNT-ITGB3i (CONi) injection (n=3 mice per group). **c-d.** Representative images and quantification analysis of Ki67 and pH3 (green) in adult mice at 14dpi after MI and AAV9-cTNT-PEX3 (CON) and/or AAV9-cTNT-ITGB3i (CONi) injection (n=6 mice per group). cTNT=Red, Hoechst=Blue. Scale bar=50 μm. (Ki67: 3814 CMs in the CON+CONi group, 3723 CMs in the CON+ITGB3i group, 3431 CMs in the PEX3+CONi group, 3707 CMs in the PEX3+ITGB3i group; pH3: 3554 CMs in the CON+CONi group, 4009 CMs in the CON+ITGB3i group, 3695 CMs in the PEX3+CONi group, 3796 CMs in the PEX3+ITGB3i group). **e.** Echocardiography measurements of ejection fraction and fractional shortening in each group mice after MI at 1dpi (n=7 mice per group). Two-way ANOVA and Tukey's Multiple Comparison Test were performed for b-e. Data shown as mean±SEM. N.S, Not Significant, **, P<0.01, ***, P<0.001.

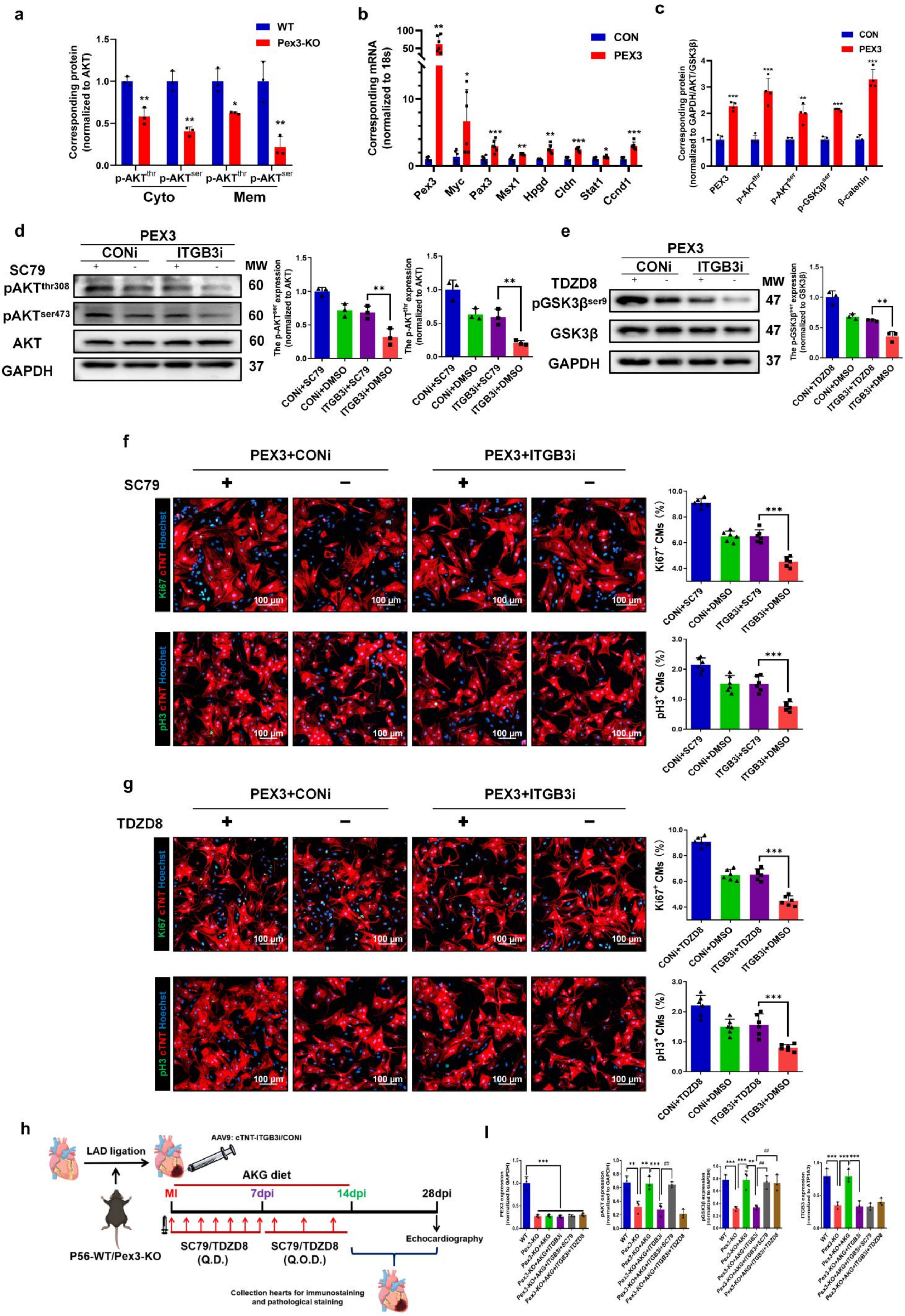
a
WT
Pex3-KO
Corresponding protein (normalized to AKT)
p-AKTthr p-AKTser p-AKTthr p-AKTser
Cyto
Mem
b
CON
PEX3
Corresponding mRNA (normalized to 18s)
Pex3 Myc Pax3 Msx1 Hpgd Cldn Stat1 Ccnd1
c
CON
PEX3
PEX3 p-AKTthr p-AKTser p-GSK3βser β-catenin
d
PEX3
CONi ITGB3i
SC79
pAKTthr308
pAKTser473
AKT
GAPDH
MW
60
60
60
37
CONi+SC79 CONi+DMSO ITGB3i+SC79 ITGB3i+DMSO
e
PEX3
CONi ITGB3i
TDZD8
pGSK3βser9
GSK3β
GAPDH
MW
47
47
37
CONi+TDZD8 CONi+DMSO ITGB3i+TDZD8 ITGB3i+DMSO
f
PEX3+CONi
PEX3+ITGB3i
SC79
Ki67 cTNT Hoechst
pH3 cTNT Hoechst
100 μm
Ki67+ CMs (%)
pH3+ CMs (%)
g
PEX3+CONi
PEX3+ITGB3i
TDZD8
h
LAD ligation
AAV9: cTNT-ITGB3i/CONi
AKG diet
MI
7dpi
14dpi
28dpi
Echocardiography
P56-WT/Pex3-KO
SC79/TDZD8 (Q.D.)
SC79/TDZD8 (Q.O.D.)
Collection hearts for immunostaining and pathological staining
I

Supplemental Figure 12: a. Quantification of AKT, p-AKT$^{thr308/ser473}$ in the cytoplasm and membrane of WT and Pex3-KO mice at 14dpi (n=3 mice per group). **b.** mRNA expression level of downstream genes in the primary cardiomyocytes after treated with Ad5-cTNT-PEX3 or Ad5-cTNT-CON. The fold change was calculated by normalizing to 18S (n=6 per group). **c.** Quantification of PEX3, p-AKT$^{thr308/ser473}$, p-GSK3β^{ser9} and β-catenin in primary cardiomyocytes transfected with Ad5-cTNT-PEX3/CON (n=4 per group). **d**. Western blotting and quantification analysis of p-AKT^{thr308}, p-AKT^{ser473} and AKT in primary cardiomyocytes treated with Ad5-cTNT-ITGB3i (CONi) and/or SC79 after transfection with Ad5-cTNT-PEX3 (n=3 per group). **e.** Western blotting of GSK3β and p-GSK3β^{ser9} in primary cardiomyocytes treated with Ad5-cTNT-ITGB3i (CONi) and/or TDZD8 after transfection with Ad5-cTNT-PEX3 (n=3 per group). **f.** Representative images and quantification analysis of Ki67 and pH3 (green) in primary cardiomyocytes treated with Ad5-cTNT-ITGB3i (CONi) and/or SC79 after transfection with Ad5-cTNT-PEX3 (n=6 per group). cTNT=Red, Hoechst=Blue. Scale bar=100 μm. (Ki67: 3362 CMs in the PEX3+CONi+SC79 group, 3347 CMs in the PEX3+CONi+DMSO group, 3056 CMs in the PEX3+ITGB3i+SC79 group, 2986 CMs in the PEX3+ITGB3i+DMSO group; pH3: 2763 CMs in the PEX3+CONi+SC79 group, 2800 CMs in the PEX3+CONi+DMSO group, 3274 CMs in the PEX3+ITGB3i+SC79 group, 3265 CMs in the PEX3+ITGB3i+DMSO group). **g.** Representative images and quantification analysis of Ki67 and pH3 (green) in primary cardiomyocytes treated with Ad5-cTNT-ITGB3i (CONi) and/or TDZD8 after transfection with Ad5-cTNT-PEX3 (n=6 per group). cTNT=Red, Hoechst=Blue. Scale bar=100 μm. (Ki67: 3107 CMs in the PEX3+CONi+TDZD8 group, 3099 CMs in the PEX3+CONi+DMSO group, 3245 CMs in the PEX3+ITGB3i+TDZD8 group, 2899 CMs in the PEX3+ITGB3i+DMSO group; pH3: 2878 CMs in the PEX3+CONi+TDZD8 group, 2773 CMs in the PEX3+CONi+DMSO group, 2946 CMs in the PEX3+ITGB3i+TDZD8 group, 3071 CMs in the PEX3+ITGB3i+DMSO group). **h.** Schematic illustration of the experimental design. **i.** Quantification of PEX3, p-AKT, p-GSK3β^{ser9} and ITGB3 in Pex3-KO mice after MI and treated with AAV9-cTNT-ITGB3i, AKG, SC79 or TDZD8 at 14dpi (n=3 mice per group). Unpaired t test applied for a-c. Two-way ANOVA and Tukey's Multiple Comparison Test were performed for d-g. One-way ANOVA and Tukey's Multiple Comparison Test were performed for i. Data shown as mean±SEM. *, P<0.05, **, P<0.01, ***, P<0.001. Pex3-KO + AKG + ITGB3i vs. Pex3-KO + AKG + ITGB3i + SC79/TDZD8, ##, P<0.01.

Supplemental Tables

Supplemental Table 1. Biological functions of peroxins family members.

Peroxins	Biological function
Pex1[1]	Mediating the fusion of pre-peroxisome vesicles during peroxisome generation
Pex2[2]	Forming a ring complex with Pex10 and Pex12
Pex3[3]	Docking with receptors Pex19 and Pex14 to form the complex required for starting the production process of peroxidase; Binding to Atg30 and mediating autophagic clearance of peroxisomes

Pex5[4]	Recognizing PTS signals and mediating the input of peroxisome matrix proteins
Pex6[1]	Mediating the fusion of peroxisome precursor vesicles
Pex7[5]	Recognizing PTST2 signaling and mediating the entry of substances with PTST2 signaling into peroxisomes
Pex10[6]	Forming a ring complex with Pex2 and Pex12
Pex11[7]	Participating in peroxisome membrane lengthening, peroxisome division and proliferation
Pex12[2]	Forming a ring complex with Pex2 and Pex10
Pex13[8]	Involved in the formation of receptor recognition complexes
Pex14[9]	Involved in the formation of receptor recognition complexes
Pex19[3]	Targeting localization of PMPs, recognition of Pex3 on the membrane, constituting the original vesicles
Pex26[10]	Membrane receptor of Pex1 and Pex6

Supplemental Table 2. Top 15 in network string_interactions_short.tsv ranked by MNC method.

Rank	Name	Score
1	Itgb3	12
2	Cd44	11
3	Itga6	10
4	Itga5	7
5	Itga2b	6
5	Cd34	6

5	Itga9	6
8	Calr	5
8	Thbs1	5
8	Hspa5	5
8	Actn4	5
8	Anpep	5
8	Tln2	5
14	Pdia6	4
14	Prkca	4

Supplemental Table 3. The sequences of qRT-PCR primers.

**Primer (mouse)**	**Sequence (5' to 3')**
18S-F	TAACGAACGAGACTCTGGCAT
18S-R	CGGACATCTAAGGGCATCACAG
Pex1-F	AAGGAAGAGCGTATTAAGCTGGA
Pex1-R	TCGATTTCCGCACTCTGTTCT
Pex2-F	AGTCAGTTGGATGCACTTGAAC
Pex2-R	TGGCTGGTATATCAGGTTGAGAG
Pex3-F	GCTCGGCGACAGTACCATTTT
Pex3-R	CGAGTTGAGCTGCTGCATTAAG
Pex5-F	CTGGTGGAGGGCGAATGTG
Pex5-R	GTCCTGGGTGAAATGGGTGG
Pex6-F	CAGGTGGCGGGAGATGTTTTT

Pex6-R	CAGCCAGGTATAAAGAGGTGTG
Pex7-F	CCGAGTTCTCTCCGTACCTG
Pex7-R	ACGTCAAACAAGCCGTCATTC
Pex10-F	TAGCGGGTGCAAAGAAATGG
Pex10-R	TGAGGCCAAAGTAGGCTATGT
Pex11-F	GACGCCTTCATCCGAGTCG
Pex11-R	CGGCCTCTTTGTCAGCTTTAGA
Pex12-F	GCTGAGTATGGGGCTCACATC
Pex12-R	CCTTGACCACATGCTGAAGAG
Pex13-F	AACCAACACTTACAAGAGTGCC
Pex13-R	CCGTAGGCTCCATATCCAGAAG
Pex14-F	ACAGCAGTGAAGTTCCTACAGA
Pex14-R	GCCAGGTCAATCTCTTCGTCT
Pex19-F	CGATGCAAGTTCTCAGCAAGA
Pex19-R	GCCAGGCCACTTAACGTCT
Pex26-F	TCCATGCCGCACTAGAGACTT
Pex26-R	GAACCTGGTAGTACCGGAGGA
Pmp70-F	GGTGCTGGAGAAATCATCA
Pmp70-R	CCAAGAACACGGAAGAGG
Gsto1-F	GCCCGAGTGGTTCTTTG
Gsto1-R	TTGTACGGGTCATCTGGAA
Nqo1-F	CTCGTAGCAGGATTTGCC
Nqo1-R	GAAGCCACAGAAACGCA

Hmox1-F	ACAGCCCCACCAAGTTC
Hmox1-R	GGCGGTCTTAGCCTCTTC
Cat-F	CCTCGCAGAGACCTGATG
Cat-R	GCACCTGCTCCTTTTGC
Nfe2l2-F	GATGGACTTGGAGTTGCC
Nfe2l2-R	CCTTCTGGAGTTGCTCTTG
Agps-F	TGGCTGCTGGAGAAGATAA
Agps-R	ATAACCCTGTCCCAAGGAG
Gnpat-F	CGCAGCTACATTGACTTCC
Gnpat-R	CTGACCACTCTCATTCCCA
Myc-F	ATGCCCCTCAACGTGAACTTC
Myc-R	CGCAACATAGGATGGAGAGCA
Pax3-F	CCGGGGCAGAATTACCCAC
Pax3-R	GCCGTTGATAAATACTCCTCCG
Msx1-F	TGCTGCTATGACTTCTTTGCC
Msx1-R	GCTTCCTGTGATCGGCCAT
Hpgd-F	GTGAACGGCAAAGTGGCTCT
Hpgd-R	TCCAATCCACCAATGCTACCT
Cldn-F	GGGGACAACATCGTGACCG
Cldn-R	AGGAGTCGAAGACTTTGCACT
Stat1-F	TCACAGTGGTTCGAGCTTCAG
Stat1-R	GCAAACGAGACATCATAGGCA

Ccnd1-F	GCGTACCCTGACACCAATCTC
Ccnd1-R	CTCCTCTTCGCACTTCTGCTC

Supplemental Table 4. Immunofluorescence, heart weight, body weight, heart weight/body weight and echocardiographic values in Figure 2b-i and Supplemental Figure 3e-f.

Parameter	WT	Pex3-KO	P-value (WT vs Pex3-KO)
Mice(n)	6	6	
P1 Ki67$^+$ CMs (%)	12.529±0.223	12.361±0.271	0.9668
P3 Ki67$^+$ CMs (%)	8.617±0.214	7.646±0.319	0.0011
P7 Ki67$^+$ CMs (%)	3.607±0.113	1.423±0.069	<0.001
P14 Ki67$^+$ CMs (%)	1.060±0.077	0.537±0.044	0.1737
P28 Ki67$^+$ CMs (%)	0.989±0.047	0.504±0.033	0.2386
P1 pH3$^+$ CMs (%)	2.465±0.141	2.434±0.106	0.9996
P3 pH3$^+$ CMs (%)	2.003±0.126	1.565±0.100	0.0017
P7 pH3$^+$ CMs (%)	1.053±0.059	0.493±0.047	<0.001
P14 pH3$^+$ CMs (%)	0.265±0.020	0.140±0.013	0.8033
P28 pH3$^+$ CMs (%)	0.236±0.020	0.118±0.012	0.8347
P1 Mononucleated CMs (%)	93.050±0.942	93.035±0.873	>0.999
P3 Mononucleated CMs (%)	87.651±0.819	84.612±0.693	0.033
P7 Mononucleated CMs (%)	75.832±0.796	71.898±0.511	0.003
P14 Mononucleated CMs (%)	65.892±0.828	62.391±0.519	0.010

P28 Mononucleated CMs (%)	64.088±0.831	60.634±0.659	0.011
P1 Bi-/multinucleated CMs (%)	6.950±0.942	6.965±0.873	>0.999
P3 Bi-/multinucleated CMs (%)	12.349±0.819	15.388±0.693	0.033
P7 Bi-/multinucleated CMs (%)	24.168±0.796	28.102±0.511	0.003
P14 Bi-/multinucleated CMs (%)	34.108±0.828	37.609±0.519	0.010
P28 Bi-/multinucleated CMs (%)	35.912±0.831	39.366±0.659	0.011
P1 HW/BW	6.162±0.030	6.169±0.023	>0.9999
P3 HW/BW	6.273±0.048	6.306±0.033	0.9737
P7 HW/BW	6.594±0.039	6.597±0.027	>0.9999
P14 HW/BW	6.442±0.034	6.454±0.027	0.9997
P28 HW/BW	6.498±0.052	6.475±0.034	0.9949
P1 Heart Weight (mg)	10.033±0.076	9.983±0.087	>0.9999
P3 Heart Weight (mg)	18.183±0.172	18.017±0.285	0.9997
P7 Heart Weight (mg)	24.000±0.141	23.567±0.275	0.9749
P14 Heart Weight (mg)	35.700±0.291	32.583±0.268	<0.001
P28 Heart Weight (mg)	126.600±1.892	115.267±1.040	<0.001
P1 Body Weight (g)	1.628±0.013	1.6188±0.012	>0.9999
P3 Body Weight (g)	2.898±0.010	2.857±0.039	0.9950
P7 Body Weight (g)	3.640±0.024	3.572±0.029	0.9553
P14 Body Weight (g)	5.542±0.028	5.048±0.038	<0.001
P28 Body Weight (g)	19.488±0.164	17.802±0.100	<0.001
CMs size (um^2)	230.8±1.985	233.1±2.613	0.491

P28 Mono-nucleus CMs (%)	24.50±1.328	13.99±0.403	<0.001
P28 Bi/Multi-nucleus CMs (%)	74.22±1.053	86.02±0.366	<0.001
Mice(n)	9	9	
P1 EF (%)	80.334±1.467	80.253±1.085	>0.9999
P7 EF (%)	81.964±1.127	82.707±1.130	0.9921
P1 FS (%)	45.866±1.476	45.504±1.085	0.9992
P7 FS (%)	48.016±1.188	48.961±1.180	0.9679
P14 EF (%)	69.035±1.782	68.614±1.833	0.9991
P28 EF (%)	70.703±1.011	60.471±1.226	<0.001
P14 FS (%)	37.160±1.341	36.684±1.399	0.9976
P28 FS (%)	38.805±0.897	31.133±0.794	<0.001

Supplemental Table 5. The level of MDA and immunofluorescence values in Supplemental Figure 4e-g.

Parameter	WT	Pex3-KO	P-value (WT vs Pex3-KO)
Mice	6	6	
P1 DHE fluorescence intensity	4.042±0.069	4.077±0.033	>0.9999
P3 DHE fluorescence	4.753±0.091	5.077±0.108	0.9139

intensity			
P7 DHE fluorescence intensity	5.732±0.082	9.037±1.250	<0.001
P28 DHE fluorescence intensity	10.233±0.278	14.041±0.433	<0.001
P56 DHE fluorescence intensity	11.288±0.374	14.426±0.435	<0.001
MDA (umol/g)	0.866±0.021	1.868±0.123	<0.001
Mice	7	7	
γH2X fluorescence intensity	8.202±0.162	14.14±0.509	<0.001

Supplemental Table 6. The level of CCK-8 and CMs number, immunofluorescence and flow cytometry values in Figure 3b-j and Supplemental Figure 5e-m.

Parameter	CON	PEX3	CONi	PEX3i	P-value (CON vs PEX3)	P-value (CONi vs PEX3i)

Samples(n)	10	10	10	10		
CCK-8	1.0±0.018	1.161±0.017	1.0±0.018	0.868±0.010	<0.001	<0.001
Samples(n)	6	6	6	6		
CMs number	1534±83.29	2151±52.98	1534±74.58	1033±13.85	<0.001	<0.001
$Ki67^+$ CMs (%)	3.686±0.155	6.429±0.149	3.661±0.146	1.771±0.137	<0.001	<0.001
$pH3^+$ CMs (%)	0.751±0.056	1.562±0.097	0.709±0.047	0.324±0.035	<0.001	<0.001
Aurora B^+ CMs (%)	4.673±0.408	9.084±0.470	4.376±0.420	1.539±0.368	<0.001	<0.001
EdU^+ CMs (%)	2.387±0.136	4.156±0.148	2.386±0.114	1.225±0.070	<0.001	<0.001
$Tunel^+$ CMs (%)	8.818±0.142	5.520±0.118	8.535±0.112	11.87±0.155	<0.001	<0.001
Samples(n)	6	6	6	6		
DCF FSI	0.969±0.022	0.683±0.008	0.964±0.016	2.021±0.130	<0.001	<0.001
Samples(n)	3	3	3	3		
G1 phase CMs (%)	73.640±0.250	64.500±0.508	71.040±0.113	79.033±0.223	<0.001	<0.001
S phase CMs (%)	9.997±0.192	14.240±0.490	16.287±0.108	11.467±0.048	0.0013	<0.001

G2 phase CMs (%)	16.363±0.227	21.260±0.139	12.670±0.064	9.500±0.249	<0.001	<0.001

Supplemental Table 7. Immunofluorescence, the level of MDA, heart weight/body weight, echocardiographic and masson values in Figure 4c-n.

Parameter	WT	Pex3-KO	P-value (WT vs Pex3-KO)
Mice(n)	6	6	
$Ki67^{+}$ CMs (%)	6.883±0.220	3.441±0.117	<0.001
$pH3^{+}$ CMs (%)	2.219±0.078	1.071±0.071	<0.001
Aurora B^{+} CMs (%)	0.958±0.069	0.405±0.034	<0.001
EdU^{+} CMs (%)	8.526±0.196	4.128±0.156	<0.001
Mononucleated CMs (%)	79.809±0.620	76.311±0.555	0.002
Binucleated CMs (%)	14.411±0.474	17.311±0.500	0.002
Multinucleated CMs (%)	5.780±0.268	6.378±0.167	0.087
Samples(n)	6	6	
CMs Size (um^2)	261.6±2.510	260.3±2.782	0.732
HW/BW (mg/g)	6.073±0.046	6.057±0.043	0.803
Mice(n)	10	9	
EF 1dpr (%)	80.58±0.921	82.29±1.586	0.612

FS 1dpr (%)	46.77±0.885	48.78±1.634	0.449
EF 28dpr (%)	61.35±0.261	56.79±0.816	<0.001
FS 28dpr (%)	32.45±0.184	29.55±0.578	<0.001
△EF (%)	-19.22±1.078	-25.50±1.060	<0.001
△FS (%)	-14.32±0.996	-19.23±1.243	<0.001
Mice(n)	8	8	
Scar area (%)	0.197±0.011	2.080±0.123	<0.001

Supplemental Table 8. Immunofluorescence values in Supplemental Figure7a, 7c-e.

Parameter	CON	PEX3	P-value (CON vs PEX3)
Mice(n)	6	6	
$Flag^+$ CMs (%)	0.000±0.000	39.87±1.394	<0.001
BZ $Ki67^+$ CMs (%)	0.954±0.053	2.197±0.084	<0.001
NZ $Ki67^+$ CMs (%)	0.952±0.062	2.160±0.082	<0.001
BZ $pH3^+$ CMs (%)	0.314±0.023	0.595±0.028	<0.001
NZ $pH3^+$ CMs (%)	0.327±0.026	0.593±0.030	<0.001
BZ Aurora B^+ CMs (%)	0.185±0.041	0.418±0.025	<0.001
NZ Aurora B^+ CMs (%)	0.179±0.039	0.417±0.030	<0.001

Supplemental Table 9. Immunofluorescence, the level of MDA, echocardiographic and masson values

in Figure 5c-n and Supplemental Figure 8a-g.

Parameter	CON	PEX3	WT	Pex3-KO	P-value (CON vs PEX3)	P-value (WT vs Pex3-KO)
Mice(n)	6	6	6	6		
BZ Ki67+ CMs (%)	0.993±0.075	3.433±0.127	1.014±0.061	0.646±0.036	<0.001	<0.001
NZ Ki67+ CMs (%)	0.983±0.090	1.008±0.073			0.834	
BZ pH3+ CMs (%)	0.278±0.032	0.894±0.047	0.306±0.025	0.163±0.018	<0.001	<0.001
NZ pH3+ CMs (%)	0.273±0.028	0.289±0.029			0.714	
BZ Aurora B+ CMs (%)	0.172±0.042	0.603±0.041			<0.001	
NZ Aurora B+ CMs (%)	0.166±0.028	0.165±0.029			0.972	
BZ EdU+ CMs (%)	0.475±0.040	1.640±0.098			<0.001	
NZ EdU+ CMs (%)	0.456±0.039	0.444±0.039			0.833	
Tunel+ CMs	8.813±0.218	5.799±0.242	8.992±0.129	12.83±0.223	<0.001	<0.001

(%)						
Mononucleated CMs (%)	24.32±1.080	30.20±1.416	24.44±0.652	20.69±0.726	0.008	0.003
Binucleated CMs (%)	62.25±0.559	58.78±0.714	62.15±0.832	65.93±0.798	0.003	0.008
Multinucleated CMs (%)	13.44±1.246	11.02±1.033	13.41±0.772	13.38±1.304	0.167	0.985
MDA (umol/g)	9.648±0.984	4.350±0.493			<0.001	
DHE fluorescence intensity	16.76±0.506	13.50±0.316			<0.001	
CMs Size (um^2)	390.2±5.851	389.4±6.324	390.9±4.076	389.8±4.205	0.922	0.857
Mice(n)	7	7				
γH2X fluorescence intensity	1.000±0.077	0.496±0.074			<0.001	
Mice(n)	8	8	8	8		
EF 7dpi (%)	55.56±0.759	54.21±1.456			0.422	
FS 7dpi (%)	28.60±0.541	27.72±0.906			0.415	
EF 28dpi (%)	43.92±1.861	56.30±1.234	46.68±1.075	37.71±1.089	<0.001	<0.001

FS 28dpi (%)	21.51±1.088	29.09±0.759	23.32±0.587	18.20±0.520	<0.001	<0.001
△EF (%)	-11.65±2.515	2.09±1.509			<0.001	
△FS (%)	-7.092±1.556	1.371±0.929			<0.001	
Mice(n)	6	6	6	6		
Scar area (%)	16.87±1.173	10.64±0.586	15.60±1.772	24.09±1.366	<0.001	0.0035

Supplemental Table 10. Immunofluorescence, echocardiographic and masson values in Figure 6e-h and Supplemental Figure 9a.

Parameter	WT +Veh	WT +AKG	Pex3-KO +Veh	Pex3-KO +AKG	P-value (WT +Veh vs Pex3-KO +Veh)	P-value (Pex3-KO +Veh vs Pex3-KO +AKG)
Mice(n)	6	6	6	6		
$Ki67^+$ CMs (%)	1.057±0.062	1.451±0.092	0.645±0.037	0.992±0.056	0.0012	0.0060
$pH3^+$ CMs (%)	0.312±0.022	0.435±0.027	0.159±0.014	0.301±0.017	<0.001	<0.001
Scar area (%)	16.46±1.080	9.595±0.605	27.63±1.314	15.95±1.057	<0.001	<0.001
Mice(n)	7	7	7	7		
EF 1dpi (%)	53.33±1.668	52.75±1.892	53.56±1.588	54.44±1.585	0.9997	0.9820

FS 1dpi (%)	27.28±1.039	26.90±1.174	27.46±1.004	27.89± 0.9486	0.9994	0.9909
EF 28dpi (%)	45.43±1.120	50.32±1.154	38.02±1.276	46.22±1.083	<0.001	<0.001
FS 28dpi (%)	22.56±0.675	25.37±0.726	18.35±0.695	22.86±0.663	0.0013	<0.001

Supplemental Table 11. Immunofluorescence values in Supplemental Figure 10e-f.

Parameter	ITGB3i	CON	ITGB3	P-value (CON vs ITGB3i)	P-value (CON vs ITGB3)
Mice(n)	6	6	6		
PMP70 average signal value	0.969±0.060	1.000±0.058	1.030±0.068	0.9342	0.9392
PEX3 average signal value	1.025±0.044	1.000±0.054	1.056±0.062	0.9424	0.7516

Supplemental Table 12. Immunofluorescence values in Supplemental Figure 11c-d.

Parameter	CON	CON	Pex3	Pex3	P-value (CON	P-value (Pex3

	+CONi	+ITGB3i	+CONi	+ITGB3i	+CONi vs Pex3 +CONi)	+CONi vs Pex3 +ITGB3i)
Mice(n)	6	6	6	6		
Ki67$^+$ CMs (%)	1.087±0.058	0.607±0.030	3.335±0.125	0.836±0.026	<0.001	<0.001
pH3$^+$ CMs (%)	0.342±0.021	0.150±0.013	0.892±0.044	0.255±0.022	<0.001	<0.001

Supplemental Table 13. Immunofluorescence, echocardiographic and masson values in Figure 7i-l and Supplemental Figure 11e.

Parameter	Pex3-KO	Pex3-KO +ITGB3i	Pex3-KO +AKG	Pex3-KO +ITGB3i +AKG	P-value (Pex3-KO vs Pex3-KO +AKG)	P-value (Pex3-KO +AKG vs Pex3-KO +ITGB3i+AKG)
Mice(n)	6	6	6	6		
Ki67$^+$ CMs (%)	0.704±0.049	0.246±0.046	1.013±0.055	0.706±0.029	<0.001	<0.001
pH3$^+$ CMs (%)	0.149±0.015	0.033±0.016	0.323±0.023	0.164±0.015	<0.001	<0.001

Scar area (%)	27.07±0.971	42.12±2.032	15.42±0.940	27.19±1.344	<0.001	<0.001
Mice(n)	7	7	7	7		
EF 1dpi (%)	52.05±1.106	53.60±1.368	53.37±1.722	55.01±1.973	0.9331	0.8824
FS 1dpi (%)	26.40±0.627	27.57±0.850	27.36±1.095	28.21±1.240	0.8993	0.9267
EF 28dpi (%)	38.47±1.126	32.81±0.676	47.05±1.412	38.79±0.974	<0.001	<0.001
FS 28dpi (%)	18.64±0.631	15.59±0.338	23.47±0.843	18.69±0.486	<0.001	<0.001

Supplemental Table 14. Immunofluorescence values in Supplemental Figure 12f.

Parameter	PEX3+ CONi+SC79	PEX3+ CONi+DMSO	PEX3+ ITGB3i+DMSO	PEX3+ CONi+SC79	P-value (PEX3+ ITGB3i+DMSO vs PEX3+ ITGB3i+SC79)
Samples(n)	6	6	6	6	
Ki67+ CMs (%)	9.094±0.134	6.491±0.167	6.505±0.202	4.524±0.154	<0.001
pH3+ CMs (%)	2.153±0.092	1.515±0.112	1.511±0.105	0.761±0.061	<0.001

Supplemental Table 15. Immunofluorescence values in Supplemental Figure 12g.

Parameter	PEX3+ CONi+TDZD8	PEX3+ CONi+DMSO	PEX3+ ITGB3i+DMSO	PEX3+ CONi+ TDZD8	P-value (PEX3+ ITGB3i+DMSO vs PEX3+ CONi+ TDZD8)
Samples(n)	6	6	6	6	
$Ki67^{+}$ CMs (%)	9.094±0.146	6.493±0.175	6.536±0.178	4.487±0.154	<0.001
$pH3^{+}$ CMs (%)	2.203±0.142	1.497±0.105	1.567±0.148	0.799±0.045	<0.001

Supplemental Table 16. Immunofluorescence, echocardiographic and masson values in Figure 8f-i.

Parameter	Mice	$Ki67^{+}$ CMs (%)	$pH3^{+}$ CMs (%)	Scar area (%)	Mice	EF (%)	FS (%)
WT (a)	6	1.024±0.048	0.315±0.019	17.46±0.819	7	45.20±0.945	22.21±0.523
Pex3-KO (b)	6	0.649±0.039	0.154±0.014	26.44±1.760	7	38.09±0.988	18.13±0.538
Pex3-KO +AKG (c)	6	0.995±0.057	0.329±0.024	14.80±1.299	7	46.43±1.120	22.95±0.649
Pex3-KO +AKG +ITGB3i (d)	6	0.631±0.035	0.161±0.013	25.03±1.766	7	38.48±0.798	18.46±0.442

Pex3-KO +AKG +ITGB3i+SC79 (e)	6	1.058±0.045	0.332±0.025	13.31±0.462	7	47.78±0.893	23.78±0.483
Pex3-KO +AKG +ITGB3i+TDZD8 (f)	6	1.074±0.058	0.335±0.019	14.00±0.843	7	47.24±0.819	23.48±0.498
P-value (a vs b)		<0.001	<0.001	<0.001		<0.001	<0.001
P-value (b vs c)		<0.001	<0.001	<0.001		<0.001	<0.001
P-value (c vs d)		<0.001	<0.001	<0.001		<0.001	<0.001
P-value (d vs e)		<0.001	<0.001	<0.001		<0.001	<0.001
P-value (d vs f)		<0.001	<0.001	<0.001		<0.001	<0.001

Supplemental References

1 Judy, R. M., Sheedy, C. J. & Gardner, B. M. Insights into the Structure and Function of the Pex1/Pex6 AAA-ATPase in Peroxisome Homeostasis. *Cells* **11**, doi:10.3390/cells11132067 (2022).

2 Platta, H. W. *et al.* Pex2 and pex12 function as protein-ubiquitin ligases in peroxisomal protein import. *Mol Cell Biol* **29**, 5505-5516, doi:10.1128/MCB.00388-09 (2009).

3 Jansen, R. L. M. & van der Klei, I. J. The peroxisome biogenesis factors Pex3 and Pex19: multitasking proteins with disputed functions. *FEBS Lett* **593**, 457-474, doi:10.1002/1873-3468.13340 (2019).

4 Skowyra, M. L. & Rapoport, T. A. PEX5 translocation into and out of peroxisomes drives matrix protein import. *Mol Cell* **82**, 3209-3225 e3207, doi:10.1016/j.molcel.2022.07.004 (2022).

5 Shin, J. H., Fu, T. & Kim, K. S. Pex7 selectively imports PTS2 target proteins to peroxisomes and is required for anthracnose disease development in Colletotrichum scovillei. *Fungal Genet Biol* **157**, 103636, doi:10.1016/j.fgb.2021.103636 (2021).

6 Prestele, J. *et al.* Different functions of the C3HC4 zinc RING finger peroxins PEX10, PEX2, and PEX12 in peroxisome formation and matrix protein import. *Proc Natl Acad Sci U S A* **107**, 14915-14920, doi:10.1073/pnas.1009174107 (2010).

7 Thoms, S. & Erdmann, R. Dynamin-related proteins and Pex11 proteins in peroxisome division and proliferation.

FEBS J **272**, 5169-5181, doi:10.1111/j.1742-4658.2005.04939.x (2005).

8 Rishi, G. *et al.* Hepatocyte-specific deletion of peroxisomal protein PEX13 results in disrupted iron homeostasis. *Biochim Biophys Acta Mol Basis Dis* **1866**, 165882, doi:10.1016/j.bbadis.2020.165882 (2020).

9 Zutphen, T., Veenhuis, M. & van der Klei, I. J. Pex14 is the sole component of the peroxisomal translocon that is required for pexophagy. *Autophagy* **4**, 63-66, doi:10.4161/auto.5076 (2008).

10 Guder, P. *et al.* Isoform-specific domain organization determines conformation and function of the peroxisomal biogenesis factor PEX26. *Biochim Biophys Acta Mol Cell Res* **1866**, 518-531, doi:10.1016/j.bbamcr.2018.10.013 (2019).
